# Expression of Inflammation-Related Genes Is Altered in Gastric Tissue of Patients with Advanced Stages of NAFLD

**DOI:** 10.1155/2013/684237

**Published:** 2013-03-30

**Authors:** Rohini Mehta, Aybike Birerdinc, Arpan Neupane, Amirhossein Shamsaddini, Arian Afendy, Hazem Elariny, Vikas Chandhoke, Ancha Baranova, Zobair M. Younossi

**Affiliations:** ^1^Betty and Guy Beatty Obesity and Liver Program, Inova Health System, Falls Church, VA 22042, USA; ^2^Center for the Study of Chronic Metabolic Diseases, School of Systems Biology, College of Science, George Mason University, Fairfax, VA 22030, USA; ^3^Center for Liver Diseases and Department of Medicine, Inova Fairfax Hospital, Falls Church, VA 22042, USA

## Abstract

Obesity is associated with chronic low-grade inflammation perpetuated by visceral adipose. Other organs, particularly stomach and intestine, may also overproduce proinflammatory molecules. We examined the gene expression patterns in gastric tissue of morbidly obese patients with nonalcoholic fatty liver disease (NAFLD) and compared the changes in gene expression in different histological forms of NAFLD. Stomach tissue samples from 20 morbidly obese NAFLD patients who were undergoing sleeve gastrectomy were profiled using qPCR for 84 genes encoding inflammatory cytokines, chemokines, their receptors, and other components of inflammatory cascades. Interleukin 8 receptor-beta (*IL8RB*) gene overexpression in gastric tissue was correlated with the presence of hepatic steatosis, hepatic fibrosis, and histologic diagnosis of nonalcoholic steatohepatitis (NASH). Expression levels of soluble interleukin 1 receptor antagonist (*IL1RN*) were correlated with the presence of NASH and hepatic fibrosis. mRNA levels of interleukin 8 (*IL8*), chemokine (C-C motif) ligand 4 (*CCL4*), and its receptor chemokine (C-C motif) receptor type 5 (*CCR5*) showed a significant increase in patients with advanced hepatic inflammation and were correlated with the severity of the hepatic inflammation. The results of our study suggest that changes in expression patterns for inflammatory molecule encoding genes within gastric tissue may contribute to the pathogenesis of obesity-related NAFLD.

## 1. Background

Obesity is a multisystem disorder characterized by an excessive increase in the adipose tissue. Biochemically, obesity can be defined as a failure of the normal energy homeostasis mechanisms which are required to balance the intake and the expenditure of energy [1, 2]. The regulation of the size of fat stores is a complex process and involves both central and peripheral tissues [1, 3] and over 50 secreted molecules, such as the adipocytic hormones leptin and adiponectin [4, 5], gastric ghrelin [6, 7], and intestinal cholecystokinin [8]. Many of these molecules also play a role in various diseases associated with obesity, particularly, nonalcoholic fatty liver disease (NAFLD) [7, 9].

Nonalcoholic fatty liver disease (NAFLD) is a spectrum of diseases ranging from relatively benign fatty liver (simple steatosis) to nonalcoholic steatohepatitis, or NASH, characterized by inflammation and ballooning degeneration of hepatocytes, which may progress to fibrosis or cirrhosis. NAFLD is considered to be the hepatic manifestation of metabolic syndrome affecting both adults and children [10, 11] and is thought to reach a prevalence of up to 30% in the general population [11–13]. The association of NAFLD with obesity, particularly visceral obesity, has long been recognized [12]. Although a number of pathways, such as enhanced oxidative stress, increased susceptibility to apoptosis, and insulin resistance have been implicated in the pathogenesis of NAFLD [11], little is known about the triggers of the progression to NASH, hepatic fibrosis, and ultimately cirrhosis. Not all individuals with NAFLD progress to cirrhosis. Additionally, not all obese patients develop NASH. One explanation for this differential progression maybe the contribution of nonadipose peripheral tissues to the pathogenesis of obesity-related NAFLD. Given that the stomach is one of the central organs of the digestive tract relaying satiety signals to the hypothalamus [14, 15] and is a source of peptides with critical roles in energy homeostasis (ghrelin), its participation in the development of obesity related NAFLD or its progression looks plausible. The discovery of ghrelin and its role in human metabolism has intensified the studies of hypothalamic control of the appetite and its contribution to obesity [16]. In 2005, it was found that the ghrelin-encoding gene also encodes obestatin, which, unlike ghrelin, is involved in appetite suppression [17]. In addition to ghrelin and obestatin, the stomach is the second largest source, after adipose tissue, of the appetite inhibiting peptide leptin [18–20]. Yet, studies on the role of gastric tissue in obesity-related disorders, such as NAFLD, are scarce.

In our previous study, we showed that the serum levels for common stomach hormones are altered in patients with advanced stages of NAFLD [7]. In particular, concentrations of des-acylghrelin in serum of patients with NASH were increased twofold as compared to BMI-matched controls with simple steatosis, while concentrations of ghrelin and obestatin were increased in patients with advanced liver fibrosis [7]. Other studies showed that the levels of ghrelin are related to inflammation and reduce the severity of inflammation [21, 22]. An overproduction of the ghrelin in the patients with advanced stages of chronic liver disease may be a compensatory event or a reflection of local inflammatory responses on site of their production.

Observations listed above prompted us to hypothesize that the gastric tissue in obese subjects is actively contributing to the systemic inflammation and pathogenesis of one of the complications of obesity, NAFLD. To investigate this, we performed comparative expression profiling for 84 genes encoding inflammatory cytokines, chemokines, their receptors and other components of inflammatory cascades in samples of gastric tissue removed during sleeve gastrectomy.

## 2. Methods

### 2.1. Samples

This study was approved by Inova Institutional Review Board (Federal Assurance FWA00000573). After informed consent, 20 morbidly obese NAFLD patients undergoing laparoscopic sleeve gastrectomy were included. For each patient, a large number of clinical and laboratory variables were available. Other chronic liver diseases were excluded by negative serology for hepatitis B and C, no history of toxic exposure and no other cause of chronic liver disease. Excessive alcohol consumption (>10 grams/day in women and >20 grams/day in men) was also excluded. No patients were receiving thiazolidinediones (TZDs) or medications for gastritis, including proton pump inhibitors.

From each patient, a discarded gastric tissue during sleeve gastrectomy was obtained and snap frozen with liquid nitrogen. Every gastric sample was also evaluated histologically for the presence of gastritis. As noted, samples were flash frozen in liquid nitrogen, placed in −80°C. Gene expression profiling experiments were performed using fundic samples collected from the remaining sleeve gastrectomy specimens. Samples were profiled for expression levels of 84 genes encoding inflammatory cytokines, chemokines, their receptors, and other components of inflammatory cascades using RT² Profiler PCR Arrays (Qiagen, USA) (see Supplementary Table 1 in Supplementary Material available online at http://dx.doi.org/10.1155/2013/684237).

For each patient, a liver biopsy was performed and read by the hepatopathologist. Before histopathological evaluation, each liver biopsy specimen was formalin-fixed, sectioned, and stained with hematoxylin-eosin and Masson's trichrome. The slides were reviewed following a predetermined histologic grading system; the extent of steatosis was graded as an estimate of the percentage of tissue occupied by fat vacuoles as follows: 0 = none, 1 ≤ 5%, 2 = 6–33%, 3 = 34–66%, and 4 ≥ 66%. Other histological features evaluated in H & E sections included portal inflammation, lymphoplasmacytic lobular inflammation, polymorphonuclear lobular inflammation, Kupffer cell hypertrophy, apoptotic bodies, focal parenchymal necrosis, glycogen nuclei, hepatocellular ballooning, and Mallory-Denk bodies. Patients who had hepatic steatosis (with or without nonspecific inflammation) or NASH were considered to have NAFLD. NASH was defined as steatosis, lobular inflammation, and ballooning degeneration with or without Mallory-Denk bodies and with or without fibrosis. Hepatic inflammation was defined according to an extent of immune cell infiltration (lymphoplasmacytic cells, polymorphonuclear cells, and Kupffer cell hypertrophy). For each category, score was assigned based on the following system: 0 = none, 1 = few, 2 = moderate, and 3 = many. Severity of total hepatic inflammation was determined based on the sum of the individual scores with advanced hepatic inflammation ≥3 and mild/no hepatic inflammation <3. Severity of pericellular and portal fibrosis was determined based on a similar scoring system as follows: 0 = none, 1 = mild, 2 = moderate, and 3 = marked fibrosis. Severity of total hepatic fibrosis was determined based on the sum of the individual scores (pericellular and portal fibrosis) with a score of ≥3 being considered as advanced hepatic fibrosis and a score of <3 being considered as mild/no hepatic fibrosis.

### 2.2. RNA Extraction and Reverse Transcription

Total RNA was extracted from fundic gastric tissue samples (*N* = 20) using RNeasy kit (Qiagen, USA) according to manufacturer's instructions. To determine the quantity and purity of the extracted RNA, absorbances were measured at 260 nm (A260) and 280 nm (A280) by the GeneQuant1300 spectrophotometer (GE Healthcare, USA). RNA of A260/A280 ratio of 1.8–2.1 was considered of high purity. RNA integrity was confirmed by gel electrophoresis using 1% agarose with ethidium bromide. RNA with sharp, clear 28S and 18S ribosomal RNA (rRNA) bands and the intensity of 28S rRNA band approximately twice as intense as the 18S rRNA band were used as parameters to evaluate the integrity of total RNA. 560 ng of extracted total RNA was reverse transcribed using RT^2^ first strand kit (Qiagen, USA). According to manufacturer's protocol, total RNA was treated to eliminate genomic DNA. Both random hexamers and oligo-dT primers were used to prime reverse transcription performed as recommended by enzyme manufacturer (Qiagen, USA).

### 2.3. Quantitative Real Time PCR Analysis

Quantitative real-time PCR was performed in 96 well PCR format using Bio-Rad CFX96 Real Time System (BioRad Laboratories, USA) with a ramp speed of 1°C/sec. Inflammatory cytokines and receptor RT² Profiler PCR Arrays (Qiagen, USA) were used to simultaneously examine the mRNA levels of 84 genes encoding for inflammatory cytokines, their receptors and intracellular components of inflammatory cascades along with five housekeeping genes following the manufacturer's protocol. The real-time PCR mixtures consisted of 1 *μ*L cDNA and 7.5 *μ*L of RT PCR master mix (Qiagen, USA) in a final volume of 25 *μ*L. The thermal profile of the RT-PCR procedure was repeated for 50 cycles: (1) 95°C for 10 min; (2) 10 s denaturation at 95°C and 15 s annealing at 60°C (amplification data collected at the end of each amplification step); (3) dissociation curve consisting of 10 s incubation at 95°C, 5 s incubation at 65°C, and a ramp up to 95°C (Bio-Rad CFX96 Real Time System, USA). Melt curves were used to validate product specificity.

The results of the RT² Profiler PCR Array were further confirmed by independent qPCR experiments. For the genes with significantly altered expression levels, the primers were designed using Primer3 from NCBI ([23] (Supplementary Table 2). The validation was carried out using the thermal profile for 40 cycles: (1) 95°C for 10 min; (2) 10 s denaturation at 95°C and 15 s annealing at 60°C (amplification data collected at the end of each amplification step); (3) dissociation curve consisting of 10 s incubation at 95°C, 5 s incubation at 65°C, and a ramp up to 95°C (Bio-Rad CFX96 Real Time System, USA). The real-time PCR mixtures consisted of 1 *μ*L cDNA, 5 *μ*L of SsoFast EvaGreen Supermix (Bio-Rad, USA), and 250 nM final concentration of primers (Invitrogen, USA) in a final volume of 10 *μ*L.

### 2.4. Analysis of Gene Expression Profiles

The gene expression data were presented as relative gene expression data [24]. Values were collected for the threshold cycle (*C*_*t*_) for each gene, and only *C*_*t*_ values less than 40 were considered for further analysis. Normalization of each target gene was carried out relative to five housekeeping genes [24, 25] according to the manufacturer's instructions (Qiagen, USA). Average of *C*_*t*_ values for five housekeeping genes (*C*_*t*_^AVG HKG^) on the same array (*B2M, HPRT1, RPL13A, GAPD,* and *ACTB*) was calculated. The normalized Δ*C*_*t*_ was log transformed; resultant values were utilized for calculation of the fold change of each target gene in different cohorts. For each target gene, the fold change was used to compare the gene expression levels in two different groups within a cohort (group A and group B). In this study, group A may be the diseased state and group B the nondiseased state; group A may be the advanced diseased state and group B the mild/nondiseased state.

*C*_*t*_ values of control wells (genomic DNA control, reverse transcriptase control, and positive PCR control) were examined separately for assessing the quality of each run and interpolate variability. For the validation of the PCR array results, we carried out the normalization procedure using previously validated housekeeping genes [26]. The relative gene expression values were calculated as described above.

### 2.5. Statistical Analysis

This study aimed for uncovering changes in gene expression in the stomach of patients with more advanced forms of NAFLD as compared to these with less advanced forms. Comparisons were performed for the following paired cohorts:

1. mild or no hepatic inflammation versus advanced hepatic inflammation;
2. mild steatosis versus advanced steatosis;
3. histologic NASH versus NAFLD without histologic NASH;
4. hepatic fibrosis versus NAFLD without hepatic fibrosis.

To assess the significance of gene expression differences between compared groups, univariate analyses were performed using the nonparametric Mann-Whitney test. To determine whether two variables covary, and to measure the strength of any relationship, Spearman's coefficient of correlation was used. The independent effect of significant variables (*P* ≤ 0.05) on advanced inflammation, NASH, and steatosis was assessed using multiple stepwise regression analysis with both the backward and forward stepwise selection procedures. The multiple test corrections were carried out using Benjamini-Hochberg-Yekutieli procedure that controls the false discovery rate under positive dependence assumptions reflecting known phenomenon of cocorrelation of expression levels for genes involved in the same cellular or organismal process. In case the positive dependent assumption would turn incorrect, assumption-free Benjamini-Hochberg procedure was also applied. Both procedures were executed using Bioconductor. To put our finding into perspective, both Benjamini-Hochberg-Yekutieli approved *P*values and the results of Benjamini-Hochberg test were reported.

## 3. Results

Clinical and demographic data summarized in Table 1. All the patients were obese with histologically proven NAFLD.

### 3.1. Gene Expression Differences between Patients with Mild and Advanced Hepatic Inflammation

When cohorts with mild (score < 3) and advanced hepatic inflammation (score ≥ 3) were compared, expression levels for chemokine (C-C motif) ligand 4 (*CCL4*), chemokine (C-C motif) receptor 5 (*CCR5*), chemokine (C-X-C motif) ligand 2 (*CXCL2*), chemokine (C-X-C motif) ligand 6 (*CXCL6*), interferon *α*2 (*IFNA2*), interleukin 19 (*IL19*), interleukin-1 family member 8 (*IL1F8*), and interleukin 8 (*IL8*), were significantly increased (*P* ≤ 0.05) (Table 2). Among these cytokines, *CCL4, CCR5, IFNA2, IL1F8,* and *IL8* were also independently and significantly correlated with hepatic inflammatory scores (*P* ≤ 0.05) (Table 3). Chemokine (C-C motif) ligand 21 (*CCL21*) and chemokine (C-C motif) ligand 3 (*CCL3*), on the other hand, were found to be significantly correlated (*P* ≤ 0.05) with hepatic inflammatory scores, but did not show significant differential expression in the group-wise comparisons (*P* ≥ 0.05) (Table 3).

### 3.2. Gene Expression Differences between Patients with Advanced Hepatic Steatosis and Mild or No Hepatic Steatosis

In patients with advanced hepatic steatosis (score ≥ 3), chemokine (C-X-C motif) ligand 14 (*CXCL14*), interleukin-1 family member 10 (*IL1F10*), and interleukin 8 receptor *β* (*IL8RB*) had a significant differential expression (*P* ≤ 0.05) as compared to those with mild steatosis (score ≤ 2) (Table 2). In addition, *IL8RB* and *IL1F10* levels were positively correlated with a degree of steatosis (*P* ≤ 0.05) (Table 3).

### 3.3. Gene Expression Differences between Patients with NASH and without NASH

Patients with presence of histologic NASH as compared to those NAFLD patients without NASH showed a significant differential expression of chemokine (C-C motif) receptor 3 (*CCR3*), chemokine (C-C motif) receptor 9 (*CCR9*), interleukin 1 receptor antagonist (*IL1RN*), interleukin 8 receptor *α* (*IL8RA),* and interleukin 9 (*IL9*) (*P* ≤ 0.05) (Table 2). Spearman's correlation coefficient analysis showed some of the differentially expressed genes, namely, *CCR3, CCR9, IL1RN, IL8RA,* and *IL9 *to be also positively correlated with NASH (*P* ≤ 0.05) (Table 3). Additionally, *IL8RB*, chemokine (C-X-C motif) ligand 14 (*CXCL12), *and chemokine (C-X-C motif) ligand 1 (*CCL1*) were also positively and significantly correlated with NASH (*P* ≤ 0.05) (Table 3).

### 3.4. Gene Expression Differences between Patients with and without Hepatic Fibrosis

In patients with hepatic fibrosis, only chemokine (C-X-C motif) ligand 17 (*CCL17*) was significantly upregulated (*P* ≤ 0.05) (Table 2). A different set of genes, small inducible cytokine subfamily E member 1 (SCYE1), *IL1RN,* and complement component 5 (*C5*), however, were positively correlated with severity of fibrosis (*P* ≤ 0.05) (Table 3).

### 3.5. Independent Predictors of Advanced Inflammation, NASH, and Fibrosis

To predict advanced hepatic inflammation, a single equation multivariate regression model was generated. In this model, only four variables—*CCL21, CCR5*, ALT, and age acted as predictors of advanced inflammation, where *CCL21* (*P* < 0.0007) and *CCR5* (*P* < 0.0064) were the strongest predictors (Table 4). These four predictors explain 66% of the variance in the inflammation phenotype (*R* ^2^ = 0.66).

For understanding the effect of independent variables on pathogenesis of histologic NASH, the multivariate regression generated a statistically significant model (*P* < 0.002) with *CCR3, CXCL12, IL1RN, IL8RA, IL8RB,* and interleukin 5 (*IL5*). This model explained 75% of the variance in NASH phenotype (*R* ^2^ = 0.75).

The model of advanced hepatic fibrosis (*P* < 0.006) included only *IL1RN *(*P* < 0.006) as a sole component explaining 34% of the variance in fibrosis (*R* ^2^ = 0.34). Interestingly, none of the genes showing differential regulation (*P* ≤ 0.05) or significantly correlated with the degree of steatosis were able to contribute significantly to the model for steatosis; hence, no models resulted from these analyses.

## 4. Discussion

Liver is a major organ involved in lipid metabolism. However, it has limited capacity to store lipids [27]. Therefore, excess lipid buildup can result in the development of NAFLD. One of the critical thrusts in the studies of the progression of NALFD has been the search for factors that may influence the progression of steatosis to NASH and cirrhosis. According to the multiple hit model of NAFLD, many hits may act in parallel or in tandem contributing to this pathogenesis. Of these, gut-derived and adipose tissue–derived factors potentially play an important role contributing to inflammatory conditions, including NAFLD. Inflammation, a central player in the pathogenesis of NASH, can enhance the probability of progression of fibrosis to NASH-related cirrhosis [28].

In the past decade, white adipose tissue has been considered as a major source for inflammatory cytokines and chemokines in obese patients [29–31]. In addition to the adipose tissue, it was suggested that other tissues, particularly, gastric and intestinal tissues may overproduce various soluble molecules and contribute to overall inflammatory background influencing distant organs [31].

**Figure 1:**
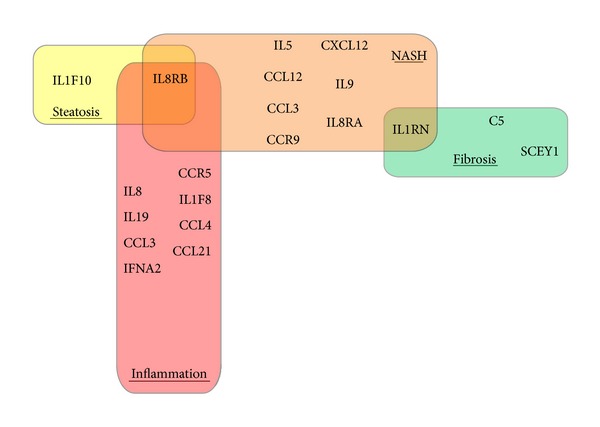
Venn diagram depicting results of an analysis of correlations. Sets of genes significantly correlating (*P* ≤ 0.05) with specific histological characteristic of nonalcoholic fatty liver disease (NAFLD) overlap only minimally.

Our study is the first to show that mRNAs encoding for various soluble molecules are overproduced in the gastric tissue of morbidly obese patients with advanced forms of NAFLD. Remarkably, there was a substantial overlap in genes with significant differential expression (*P* ≤ 0.05) and genes with significant correlation (*P* ≤ 0.05) to the same histological characteristic of NAFLD (Supplementary Figure 1). Further, distinct and notably, nonoverlapping sets of soluble molecule encoding genes change their expression along with various histological features of NAFLD (Figure 1). Importantly, an overlap between sets of genes significantly correlating (*P* ≤ 0.05) with a specific histological characteristic of NAFLD was minimal (Figure 1). *IL8RB/CXCR2* is a notable exclusion with its overexpression correlating with steatosis and diagnosis of NASH as well as fibrosis.

**Figure 2:**
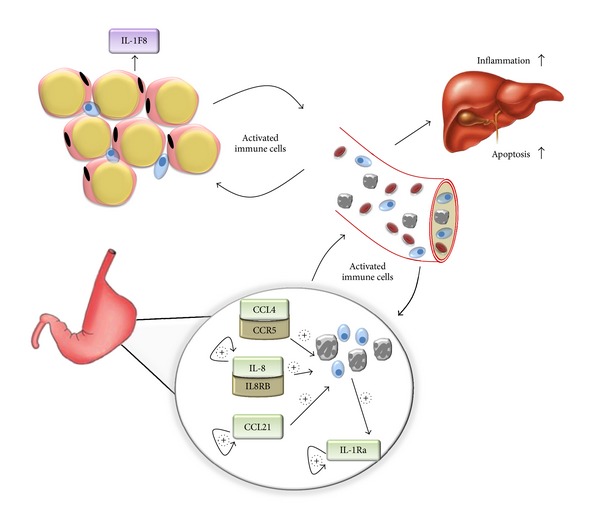
Inflammation-related genes in stomach and obesity-associated nonalcoholic fatty liver disease (NAFLD). In obesity, increased levels of inflammatory molecules such as IL1F8 may alter gene expression in stomach by activating nuclear factor kappa-light-chain-enhancer of activated B cells (NF-*κ*B). NF-*κ*B is known to activate gene expression of a number of downstream inflammatory molecules including CCL4, IL8, and CCL21. These inflammatory molecules may then regulate their own expression in positive feedback loop, thus further exacerbating inflammatory profile. These molecules can potentially activate local immune cells and attract additional immune cells. Oxidative stress triggered by activated immune cells can further add to existing inflammation. The entry of secreted inflammatory molecules and activated immune cells into portal circulation may contribute to NAFLD.

IL8RB/CXCR2 is a receptor for the IL8 chemokine that plays an important role in liver inflammation, regeneration, and repair [32, 33] as well as in the neutrophil accumulation in other inflammatory conditions [31, 34]. Increased levels of the gastric expression of *IL8RB* gene indicate that in morbidly obese patients with NASH-associated inflammation, IL8 activation is not limited to hepatic macrophages as had been shown before [32], but is a system-wide feature. It is plausible that IL8RB present on the resident gastric macrophages cells or on neutrophils activates the neutrophils locally upon its binding to IL8. In turn, activated neutrophils may then release additional chemokines and/or may enter the liver through portal circulation and influence the progression of NAFLD (Figure 2). This premise is also supported by our observation that the expression of *IL8* gene that encodes the ligand for IL8RB positively correlates with advanced hepatic inflammation (Table 3). Circulatory IL8 levels are reported to increase under oxidative stress and, in turn, stimulate further increase in levels of oxidant stress mediators by local recruitment of inflammatory cells [35] (Figure 2). As an expanding adipose tissue of obese individuals releases increased levels of IL8 [9, 30], it may trigger increased expression of gastric IL8 and its receptor IL8RB. Additionally, studies have shown that free fatty acids (FFA), also increased in obese individuals, influence expression of IL8 in various peripheral tissues [36, 37]. Thus, the paired increase in levels of IL8 and its receptor found in the gastric tissue of obese may act to activate local as well as circulating, thus contributing towards vicious cycle of inflammation and influencing progression of NAFLD.

The expression levels of anti-inflammatory receptor IL1RN, an antagonist of IL1A and IL1B, were positively correlated both with the presence of NASH and with fibrosis (Table 3). In the regression model predicting fibrosis, expression of *IL1RN* mRNA was the only significant component that explained 34% of the variance in fibrosis. Additionally, *IL1RN* mRNA expression significantly contributed to the regression model predicting NASH (Table 4). These observations are in agreement with a recent report on association of serum IL1Ra levels and liver *IL1RN* expression with NASH [38]. IL1Ra is expressed and secreted by a number of immune cells such as monocytes, macrophages, and neutrophils as well as epithelial cells and hepatocytes [39]. As its expression is regulated by proinflammatory cytokines, IL1RN is considered to be an acute phase protein [40] with levels elevated in many inflammatory conditions [41]. We hypothesize that increased levels of circulating and/or local proinflammatory cytokines upregulate gastric IL1RN expression either directly or via activated leukocytes (Figure 2). Once upregulated, IL1Ra may stimulate its own gastric expression by a positive feedback loop (Figure 2). This mechanism is supported by studies showing elevated circulating IL1RN in patients with obesity [40] and NAFLD [38, 42].

Many genes differentially expressed in the gastric tissue of patients with advanced forms of NAFLD encode chemokines previously shown as important players in a variety of inflammatory conditions. For example, expression levels of both* CCL4* chemokine and its receptor *CCR5* encoding genes showed significant upregulation in advanced hepatic inflammation (Table 2) and a positive correlation with the severity of the hepatic inflammation (*P* ≤ 0.05) (Table 3). In the multivariate regression model, *CCR5 *mRNA level also was one of the strongest predictors of the severity of hepatic inflammation (Table 4). CCL4 attracts natural killer cells, monocytes, and a variety of other immune cells [1]. The increased expression of *CCL4* and *CCR5 *genes in gastric tissue could be attributed to local immune cells activated in response to upstream regulators like IL1F8 (Figure 2). In the present study, *IL1F8* gene was also upregulated in stomach tissue of patients with advanced liver inflammation (Tables 2 and 5). CCR5 has been implicated in NASH [43] and hepatic fibrosis [44]. Both of these conditions develop almost exclusively in a proinflammatory environment. While the role of CCL4/CCR5 in the pathogenesis of NAFLD remains to be sketched out, these collective findings make it an attractive target for further investigation.

The complex interaction of cytokines, chemokines, and their receptors highlighted in this study suggests that the gastric tissue is an integral player in obesity-associated NAFLD. It seems that in obesity, an increase in inflammatory responses of adipose tissue corresponds to similar increase in the inflammation within the tissues involved in satiety response. Activated immune cells embedded in the gastric tissue may then recruit additional immune cells or be released in circulation, and hence amplify the inflammatory response and promote the development and progression of NAFLD (Figure 2). An increase in recognition of the endocrine function of the stomach and its contributions to energy homeostasis prompts us to hypothesize that its altered inflammatory profile may influence its endocrine secretion. This, in turn, may trigger a cascade of metabolic dysfunction culminating in NAFLD (Figure 2). It remains to be determined if the complex interaction of inflammatory molecules in gastric tissue lies upstream or downstream of the intricate network of inflammatory signaling, which is the hallmark of NAFLD. Evidently, the stomach plays a certain role in metabolic dysfunction; its potential proinflammatory properties should not be neglected by studies of the conditions related to metabolic syndromes, including NAFLD.

## 5. Conclusion

In this study, we demonstrate an altered pattern of gene expression for cytokine and chemokine encoding genes in the gastric tissue of individuals with obesity and varying degrees of hepatic inflammation and different forms of NAFLD. Soluble inflammatory molecules produced by the stomach appear to contribute to obesity-related NAFLD. Although the causal links between these signaling events remains to be determined, we propose that the fundus of the stomach is an integral player in the signaling milieu associated with both obesity-related NAFLD.

## Supplementary Material

Supplementary Materials contains following Tables and Figure:
Supplementary Table 1: Inflammatory cytokines and receptor encoding genes profiled for their expression levels in fundic gastric samples of 20 obese subjects.
Supplementary Table 2: Primer sequences used in validation study.
Supplementary Figure 1: A substantial overlap in genes with significant differential expression (*P* ≤ 0.05) and genes with significant correlation (*P* ≤ 0.05) to the same histological characteristics of NAFLD.

## Figures and Tables

**Table 1:** Demographic and clinical characteristics of the patient cohorts profiled for expression of inflammation- and immunity-related genes, values marked by asterisk (∗) are given as average ± SD. All subjects qualified for NAFLD had no history of alcohol abuse. No patients were taking thiazolidinediones (TZDs) or medication for gastritis.

Demographic or clinical parameter	Mean ± SD, or % (*N* = 20)
BMI (∗)	48.67 ± 8.95
AST, U/L (∗)	24.20 ± 7.13
ALT, U/L (∗)	31.25 ± 12.99
Total cholesterol, mg/dL (∗)	185.40 ± 76.61
HDL, mg/dL (∗) females	53 ± 17
HDL, mg/dL (∗) males	39.67 ± 7.09
Triglyceride, mg/dL (∗)	196.75 ± 107.28
Glucose, mg/dL (∗)	108.70 ± 36.20
Age, yr	43.44 ± 10.63
Hypertension	55% (*N* = 11)
Smoking	5% (*N* = 1)
Gender (females)	75% (*N* = 15)
Race (Caucasian)	80% (*N* = 16)
Advanced inflammation (score ≥ 3)	50% (*N* = 10)
NASH	65% (*N* = 13)
Advanced steatosis	60% (*N* = 8)
Fibrosis	75% (*N* = 15)
Steatosis with advanced inflammation	50% (*N* = 10)
NASH with advanced inflammation	30% (*N* = 6)
Gastritis	45% (*N* = 9)

SD: standard deviation; BMI: body mass index; NASH: nonalcoholic steatohepatitis; AST: aspartate aminotransferase; ALT: alanine transaminase; HDL: high-density lipoprotein.

**Table 2:** List of genes significantly upregulated in gastric tissues of patients with the following pathological conditions.

Genes	Fold change	*P* values	FDR significance (B-H pass test)
Advanced liver inflammation (score ≥ 3)/mild or no liver inflammation (score < 3)			

CCL4	2.32	0.037635	Yes
CXCL2	2.82	0.031209	Yes
CCR5	3.16	0.025748	Yes
IFNA2	3.91	0.028306	Yes
IL19	3.48	0.025748	Yes
IL1F8	4.03	0.029948	Yes
CXCL6	4.3	0.037635	Yes
IL8	4.82	0.025748	Yes

Advanced steatosis (score ≥ 3)/mild or no steatosis (score < 3)			

IL8RB	1.56	0.027891	Yes
CXCL14	1.77	0.033865	Yes
IL1F10	2.24	0.049141	Yes

NASH/No NASH^‡^			

CCR9	2.96	0.047583	Yes
CCR3	3.44	0.047583	Yes
IL1RN	3.95	0.021559	Yes
IL9	9.49	0.021363	Yes
IL8RA	15.04	0.040682	Yes

Fibrosis presence/no fibrosis^‡^			

CCL17	4.81	0.029	Yes

Advanced liver inflammation (score ≥ 3) (*N* = 10), advanced steatosis (score ≥ 3) (*N* = 8), NASH^‡^ (*N* = 13), fibrosis (*N* = 15). ^‡^Comparison was performed for groups of patients without the condition listed. NASH: nonalcoholic steatohepatitis.

**Table 3:** Correlations between inflammatory gene expression levels (dependent variable) and the following pathological conditions (independent variable).

Gene	Spearman correlation	*P* values	FDR significance (B-H pass test )
Advanced liver inflammation (score ≥ 3)			

CCL3	0.45	0.041336	Yes
CCL4	0.47	0.035439	Yes
IL8	0.45	0.042123	Yes
CCR5	0.48	0.031463	Yes
IL8RB	0.50	0.024563	Yes
CCL21	0.50	0.024304	Yes
IFNA2	0.51	0.024371	Yes
IL19	0.51	0.020672	Yes
IL1F8	0.53	0.013993	Yes

Advanced steatosis (score ≥ 3)			

IL1F10	0.45	0.043652	Yes
IL8RB	0.49	0.025506	Yes

NASH			

CXCL12	−0.44	0.049062	Yes
CCL1	0.45	0.045748	Yes
CCR3	0.46	0.039522	Yes
CCR9	0.46	0.039522	Yes
IL5	0.44	0.049062	Yes
IL8RA	0.47	0.032636	Yes
IL8RB	0.44	0.049062	Yes
IL1RN	0.53	0.014784	Yes
IL9	0.53	0.014605	Yes

Fibrosis			

C5	0.46	0.038905	Yes
SCYE1	0.48	0.030164	Yes
IL1RN	0.53	0.015503	Yes

Advanced liver inflammation (score ≥ 3) (*N* = 10), advanced steatosis (score ≥ 3) (*N* = 8), NASH (*N* = 13), fibrosis (*N* = 15). NASH: nonalcoholic steatohepatitis.

**Table 4:** Best fitting multiple linear regression models showing the relationship between predictor variables and the predicted clinical parameter.

Group	Independent variable	Regression coefficient *β*	*P* values of independent variables	*P* value of the entire model
Advanced liver inflammation (score ≥ 3)	(Intercept)	−0.5745 ± 0.8110	0.4896	
	CCL21	0.0012 ± 0.0003	0.0007	
	CCR5	0.0004 ± 0.0001	0.0064	*P* < 0.001
	AGE	0.0288 ± 0.0162	0.0964	
	ALT	0.0267 ± 0.0136	0.0688	

	(Intercept)	0.3545 ± 0.1988	0.0980	
	CCR3	0.0003 ± 0.0002	0.0626	
	CXCL12	−0.0001 ± 0.0000	0.0724	
NASH	IL1RN	0.0003 ± 0.0001	0.0810	*P* < 0.002
	IL5	−0.0003 ± 0.0001	0.0683	
	IL8RA	0.0655 ± 0.0215	0.0092	
	IL8RB	0.0004 ± 0.0002	0.0532	

Fibrosis	(Intercept)	0.5750 ± 0.2471	0.0318	*P* < 0.006
	IL1RN	0.0007 ± 0.0002	0.0063	

Regression coefficient *β* represents slope estimate ± standard error of the estimate (SE) (*P* ≤ 0.05 were considered significant).

**Table 5:** Validation of PCR array data by individual qPCR assays for the selected set of genes.

Genes	Fold change	*P* value
Advanced liver inflammation (score ≥ 3)/mild/no liver		
inflammation (score < 3)		

CCL4	1.5	0.01
CCR5	1.2	0.01
IFNA2	2.5	0.001
IL19	1.9	0.05
IL1F8	11.0	0.025

Advanced steatosis (score ≥ 3)/mild/no steatosis (score < 3 )		

IL8RB	1.9	0.03

NASH/no NASH^‡^		

IL1RN	1.19	0.03
IL9	2.0	0.02

## References

[1] Flier JS, Maratos-Flier E (1998). Obesity and the hypothalamus: novel peptides for new pathways. *Cell*.

[2] Kypreos KE, Karagiannides I, Fotiadou EH (2009). Mechanisms of obesity and related pathologiesrRole of apolipoprotein e in the development of obesity. *FEBS Journal*.

[3] Dobrin R, Zhu J, Molony C (2009). Multi-tissue coexpression networks reveal unexpected subnetworks associated with disease. *Genome Biology*.

[4] Baranova A, Gowder SJ, Schlauch K (2006). Gene expression of leptin, resistin, and adiponectin in the white adipose tissue of obese patients with non-alcoholic fatty liver disease and insulin resistance. *Obesity Surgery*.

[5] Morton GJ, Schwartz MW (2011). Leptin and the central nervous system control of glucose metabolism. *Physiological Reviews*.

[6] Gurriarán-Rodríguez U, Al-Massadi O, Roca-Rivada A (2011). Obestatin as a regulator of adipocyte metabolism and adipogenesis. *Journal of Cellular and Molecular Medicine*.

[7] Estep M, Abawi M, Jarrar M (2011). Association of obestatin, ghrelin, and inflammatory cytokines in obese patients with non-alcoholic fatty liver disease. *Obesity Surgery*.

[8] Chaudhri OB, Field BCT, Bloom SR (2008). Gastrointestinal satiety signals. *International Journal of Obesity*.

[9] Jarrar MH, Baranova A, Collantes R (2008). Adipokines and cytokines in non-alcoholic fatty liver disease. *Alimentary Pharmacology and Therapeutics*.

[10] de Alwis NMW, Day CP (2008). Non-alcoholic fatty liver diseasetThe mist gradually clears. *Journal of Hepatology*.

[11] Kim CH, Younossi ZM (2008). Nonalcoholic fatty liver disease: a manifestation of the metabolic syndrome. *Cleveland Clinic Journal of Medicine*.

[12] Machado M, Marques-Vidal P, Cortez-Pinto H (2006). Hepatic histology in obese patients undergoing bariatric surgery. *Journal of Hepatology*.

[13] Vernon G, Baranova A, Younossi ZM (2011). Systematic review: the epidemiology and natural history of non-alcoholic fatty liver disease and non-alcoholic steatohepatitis in adults. *Alimentary Pharmacology and Therapeutics*.

[14] Cummings DE, Overduin J (2007). Gastrointestinal regulation of food intake. *Journal of Clinical Investigation*.

[15] Woods SC (2004). Gastrointestinal Satiety Signals. I. An overview of gastrointestinal signals that influence food intake. *American Journal of Physiology*.

[16] Kojima M, Hosoda H, Date Y, Nakazato M, Matsuo H, Kangawa K (1999). Ghrelin is a growth-hormone-releasing acylated peptide from stomach. *Nature*.

[17] Zhang JV, Ren PG, Avsian-Kretchmer O (2005). Medicine: obestatin, a peptide encoded by the ghrelin gene, opposes ghrelin’s effects on food intake. *Science*.

[18] Cinti S, De Matteis R, Ceresi E (2001). Leptin in the human stomach. *Gut*.

[19] Cammisotto PG, Levy É, Bukowiecki LJ, Bendayan M (2010). Cross-talk between adipose and gastric leptins for the control of food intake and energy metabolism. *Progress in Histochemistry and Cytochemistry*.

[20] Cammisotto PG, Renaud C, Gingras D, Delvin E, Levy E, Bendayan M (2005). Endocrine and exocrine secretion of leptin by the gastric mucosa. *Journal of Histochemistry and Cytochemistry*.

[21] Jeffery P, McDonald V, Tippett E, McGuckin M (2011). Ghrelin in gastrointestinal disease. *Molecular and Cellular Endocrinology*.

[22] Baatar D, Patel K, Taub DD (2011). The effects of ghrelin on inflammation and the immune system. *Molecular and Cellular Endocrinology*.

[23] Ye J, Coulouris G, Zaretskaya I, Cutcutache I, Rozen S, Madden TL (2012). Primer-BLAST: a tool to design target-specific primers for polymerase chain reaction. *BMC Bioinformatics*.

[24] Schmittgen TD, Livak KJ (2008). Analyzing real-time PCR data by the comparative CT method. *Nature Protocols*.

[25] Vandesompele J, De Preter K, Pattyn F (2002). Accurate normalization of real-time quantitative RT-PCR data by geometric averaging of multiple internal control genes. *Genome Biology*.

[26] Birerdinc A, Mehta R, Stepanova M (2010). *Gastric Tissue Gene Expression Associated with Obesity-Related Non-Alcoholic Steatohepatitis (NASH)*.

[27] Petersen KF, Dufour S, Befroy D, Lehrke M, Hendler RE, Shulman GI (2005). Reversal of nonalcoholic hepatic steatosis, hepatic insulin resistance, and hyperglycemia by moderate weight reduction in patients with type 2 diabetes. *Diabetes*.

[28] Tilg H, Moschen AR (2010). Evolution of inflammation in nonalcoholic fatty liver disease: the multiple parallel hits hypothesis. *Hepatology*.

[29] Estep JM, Baranova A, Hossain N (2009). Expression of cytokine signaling genes in morbidly obese patients with non-alcoholic steatohepatitis and hepatic fibrosis. *Obesity Surgery*.

[30] Baranova A, Randhawa M, Jarrar M, Younossi ZM (2007). Adipokines and melanocortins in the hepatic manifestation of metabolic syndrome: nonalcoholic fatty liver disease. *Expert Review of Molecular Diagnostics*.

[31] Baranova A, Schlauch K, Gowder S, Collantes R, Chandhoke V, Younossi ZM (2005). Microarray technology in the study of obesity and non-alcoholic fatty liver disease. *Liver International*.

[32] Zimmermann HW, Seidler S, Gassler N (2011). Interleukin-8 is activated in patients with chronic liver diseases and associated with hepatic macrophage accumulation in human liver fibrosis. *PLoS ONE*.

[33] Kuboki S, Shin T, Huber N (2008). Hepatocyte signaling through CXC chemokine receptor-2 is detrimental to liver recovery after ischemia/reperfusion in mice. *Hepatology*.

[34] Chou RC, Kim ND, Sadik CD (2010). Lipid-cytokine-chemokine cascade drives neutrophil recruitment in a murine model of inflammatory arthritis. *Immunity*.

[35] Gómez-Quiroz L, Bucio L, Souza V (2003). Interleukin 8 response and oxidative stress in HepG2 cells treated with ethanol, acetaldehyde or lipopolysaccharide. *Hepatology Research*.

[36] Böni-Schnetzler M, Boller S, Debray S (2009). Free fatty acids induce a proinflammatory response in islets via the abundantly expressed interleukin-1 receptor I. *Endocrinology*.

[37] Andoh A, Takaya H, Araki Y, Tsujikawa T, Fujiyama Y, Bamba T (2000). Medium- and long-chain fatty acids differentially modulate interleukin-8 secretion in human fetal intestinal epithelial cells. *Journal of Nutrition*.

[38] Pihlajamäki J, Kuulasmaa T, Kaminska D (2012). Serum interleukin 1 receptor antagonist as an independent marker of non-alcoholic steatohepatitis in humans. *Journal of Hepatology*.

[39] Perrier S, Darakhshan F, Hajduch E (2006). IL-1 receptor antagonist in metabolic diseases: Dr Jekyll or Mr Hyde?. *FEBS Letters*.

[40] Gabay C, Smith MF, Eidlen D, Arend WP (1997). Interleukin 1 receptor antagonist (IL-1Ra) is an acute-phase protein. *Journal of Clinical Investigation*.

[41] Arena WP, Malyak M, Guthridge CJ, Gabay C (1998). Interleukin-1 receptor antagonist: role in biology. *Annual Review of Immunology*.

[42] Somm E, Cettour-Rose P, Asensio C (2006). Interleukin-1 receptor antagonist is upregulated during diet-induced obesity and regulates insulin sensitivity in rodents. *Diabetologia*.

[43] Beilhack A, Rockson SG (2003). Immune traffic: a functional overview.. *Lymphatic research and biology*.

[44] Bertola A, Bonnafous S, Anty R (2010). Hepatic expression patterns of inflammatory and immune response genes associated with obesity and nash in morbidly obese patients. *PLoS ONE*.

