# Supplementary material for: Expression of Inflammation-Related Genes Is Altered in Gastric Tissue of Patients with Advanced Stages of NAFLD
Source: Mediators Inflamm. 2013 Mar 30;2013:684237. doi: 10.1155/2013/684237 (PMC3626032; doi:10.1155/2013/684237)

**Supplementary Table 1.** Inflammatory cytokines and receptor encoding genes profiled for their expression levels in fundic gastric samples of 20 obese subjects

| **Gene Name** | | **Gene ID** | | **Biological Function** |
| --- | --- | --- | --- | --- |
| **Chemokine Genes and their Receptors** | | | | |
| Chemokine (C-C motif) ligand 1 | | *CCL1* | | Is a CXC subfamily of cytokine, secreted by activated T cells. Displays chemotactic activity for monocytes but not for neutrophils. It binds to the chemokine receptor CCR8. |
| Chemokine (C-C motif) ligand 11 | | *CCL11* | | Is a CC cytokine. Displays chemotactic activity for eosinophils, but not mononuclear cells or neutrophils. Binds to CCR3. |
| Chemokine (C-C motif) ligand 13 | | *CCL13* | | Is a CC cytokine with chemotactic activity for monocytes, lymphocytes, basophils and eosinophils, but not neutrophils. |
| Chemokine (C-C motif) ligand 15 | | *CCL15* | | The cytokine encoded by this gene is chemotactic for T cells and monocytes and induces N-acetyl-beta-D-glucosaminidase release in monocytes. It induces changes in intracellular calcium concentration in monocytes and is thought to act through the CCR1 receptor. |
| Chemokine (C-C motif) ligand 16 | | *CCL16* | | Shows chemotactic activity for lymphocytes and monocytes but not neutrophils. Induces a calcium flux in THP-1 cells that were desensitized by prior expression to RANTES. The expression of this gene is upregulated by IL-10. |
| Chemokine (C-C motif) ligand 17 | | *CCL17* | | Displays chemotactic activity for T lymphocytes, but not monocytes or granulocytes. The product of this gene binds to chemokine receptors CCR4 and CCR8. This chemokine plays important roles in T cell development in thymus as well as in trafficking and activation of mature T cells. |
| Chemokine (C-C motif) ligand 18 (pulmonary and activation-regulated) | | *CCL18* | | Displays chemotactic activity for naive T cells, CD4+ and CD8+ T cells and nonactivated lymphocytes, but not for monocytes or granulocytes. This chemokine attracts naive T lymphocytes toward dendritic cells and activated macrophages in lymph nodes. It may play a role in both humoral and cell-mediated immunity responses. |
| Chemokine (C-C motif) ligand 19 | | *CCL19* | | Play a role in normal lymphocyte recirculation and homing. It also plays an important role in trafficking of T cells in thymus, and in T cell and B cell migration to secondary lymphoid organs. It specifically binds to chemokine receptor CCR7. |
| Chemokine (C-C motif) ligand 2 | | *CCL2* | | A CC chemokine with chemotactic activity for monocytes and basophils but not for neutrophils or eosinophils. |
| Chemokine (C-C motif) ligand 20 | | *CCL20* | | Involved in formation and function of the mucosal lymphoid tissues by attracting lymphocytes and dendritic cells towards epithelial cells. C-terminal processed forms have been shown to be equally chemotactically active for leukocytes. |
| Chemokine (C-C motif) ligand 21 | | *CCL21* | | Chemotactic in vitro for thymocytes and activated T cells, but not for B cells, macrophages, or neutrophils. May also play a role in mediating homing of lymphocytes to secondary lymphoid organs. It is a high affinity functional ligand for chemokine receptor 7 (CCR7). |
| Chemokine (C-C motif) ligand 23 | | *CCL23* | | Chemotactic activity on resting T lymphocytes and monocytes, lower activity on neutrophils and no activity on activated T lymphocytes. |
| Chemokine (C-C motif) ligand 24 | | *CCL24* | | The cytokine encoded by this gene displays chemotactic activity on resting T lymphocytes, a minimal activity on neutrophils, and is negative on monocytes and activated T lymphocytes. Binds to CCR3. |
| Chemokine (C-C motif) ligand 25 | | *CCL25* | | Chemotactic activity for dendritic cells, thymocytes, and activated macrophages but is inactive on peripheral blood lymphocytes and neutrophils. The product of this gene binds to chemokine receptor CCR9. |
| Chemokine (C-C motif) ligand 26 | | *CCL26* | | Displays chemotactic activity for normal peripheral blood eosinophils and basophils. The product of this gene is one of three related chemokines that specifically activate chemokine receptor CCR3. |
| Chemokine (C-C motif) ligand 3 | | *CCL3* | | Also known as MIP-1α is a monokine with inflammatory and chemokinetic properties. Binds to CCR1, CCR4 and CCR5 receptors. |
| Chemokine (C-C motif) ligand 4 | | *CCL4* | | Monokine with inflammatory and chemokinetic properties. Binds to CCR5. The processed form MIP-1-beta retains the abilities to induce down-modulation of surface expression of the chemokine receptor CCR5. |
| Chemokine (C-C motif) ligand 5 | | *CCL5* | | Chemoattractant for blood monocytes, memory T-helper cells and eosinophils. Causes the release of histamine from basophils and activates eosinophils. Binds to CCR1, CCR3, CCR4 and CCR5. |
| Chemokine (C-C motif) ligand 7 | | *CCL7* | | This gene encodes monocyte chemotactic protein 3, a secreted chemokine which attracts macrophages during inflammation and metastasis. The protein is an in vivo substrate of matrix metalloproteinase 2, an enzyme which degrades components of the extracellular matrix. |
| Chemokine (C-C motif) ligand 8 | | *CCL8* | | Chemotactic factor that attracts monocytes, lymphocytes, basophils and eosinophils. May play a role in neoplasia and inflammatory host responses. |
| Chemokine (C-C motif) receptor 1 | | *CCR1* | | The ligands of this receptor include macrophage inflammatory protein 1 alpha (MIP-1 alpha), regulated on activation normal T expressed and secreted protein (RANTES), monocyte chemoattractant protein 3 (MCP-3), and myeloid progenitor inhibitory factor-1 (MPIF-1). Subsequently transduce a signal by increasing the intracellular calcium ions level. |
| Chemokine (C-C motif) receptor 2 | | *CCR2* | | This gene encodes two isoforms of a receptor for monocyte chemoattractant protein-1. Receptor mediates agonist-dependent calcium mobilization and inhibition of adenylyl cyclase. |
| Chemokine (C-C motif) receptor 3 | | *CCR3* | | Binds to eotaxin, eotaxin-3, MCP-3, MCP-4, RANTES and MIP-1 delta. Subsequently transduces a signal by increasing the intracellular calcium ions level. |
| Chemokine (C-C motif) receptor 4 | | *CCR4* | | It is a receptor for the CC chemokine - MIP-1, RANTES, TARC and MCP-1. Functions as a chemoattractant homing receptor on circulating memory lymphocytes and the activity of this receptor is mediated by G(i) proteins. |
| Chemokine (C-C motif) receptor 5 | | *CCR5* | | The ligands of this receptor include monocyte chemoattractant protein 2 (MCP-2), macrophage inflammatory protein 1 alpha (MIP-1 alpha), macrophage inflammatory protein 1 beta (MIP-1 beta) and regulated on activation normal T expressed and secreted protein (RANTES). Transduces a signal by increasing the intracellular calcium ion level. |
| Chemokine (C-C motif) receptor 6 | | *CCR6* | | The gene is preferentially expressed by immature dendritic cells and memory T cells. Binds to MIP-3-alpha/LARC and subsequently transduces a signal by increasing the intracellular calcium ions level. important for B-lineage maturation and antigen-driven B-cell differentiation, and it may regulate the migration and recruitment of dentritic and T cells during inflammatory and immunological responses. |
| Chemokine (C-C motif) receptor 7 | | *CCR7* | | Expressed in various lymphoid tissues and activates B and T lymphocytes. It has been shown to control the migration of memory T cells to inflamed tissues, as well as stimulate dendritic cell maturation. |
| Chemokine (C-C motif) receptor 8 | | *CCR8* | | Role in regulation of monocyte chemotaxis and thymic cell apoptosis. More specifically, this receptor may contribute to the proper positioning of activated T cells within the antigenic challenge sites and specialized areas of lymphoid tissues. |
| Chemokine (C-C motif) receptor 9 | | *CCR9* | | The specific ligand of this receptor is CCL25. Subsequently transduces a signal by increasing the intracellular calcium ions level. Role in the thymocytes recruitment and development. |
| Chemokine (C-X3-C motif) receptor 1 | | *CX3CR1* | | Receptor for chemokine Fractalkine involved in the adhesion and migration of leukocytes. |
| Chemokine (C-X-C motif) ligand 1 (melanoma growth stimulating activity, alpha) | | *CXCL1* | | CXC chemokine with chemotactic activity for neutrophils. May play a role in inflammation and exerts its effects on endothelial cells in an autocrine fashion. |
| Chemokine (C-X-C motif) ligand 10 | | *CXCL10* | | Stimulation of monocytes, natural killer and T-cell migration, and modulation of adhesion molecule expression. |
| Chemokine (C-X-C motif) ligand 11 | | *CXCL11* | | Encoded protein induces a chemotactic response in activated T-cells and is the dominant ligand for CXC receptor-3. Induces calcium release in activated T-cells. IFN-gamma is a potent inducer of transcription of this gene. |
| Chemokine (C-X-C motif) ligand 12 | | *CXCL12* | | Chemoattractant active on T-lymphocytes, monocytes, but not neutrophils. Activates the C-X-C chemokine receptor CXCR4 to induce a rapid and transient rise in the level of intracellular calcium ions and chemotaxis. Acts as a positive regulator of monocyte migration and a negative regulator of monocyte adhesion. |
| Chemokine (C-X-C motif) ligand 13 | | *CXCL13* | | Preferentially promotes the migration of B lymphocytes (compared to T cells and macrophages), apparently by stimulating calcium influx into, and chemotaxis of, cells expressing Burkitt's lymphoma receptor 1 (BLR-1). |
| Chemokine (C-X-C motif) ligand 14 | | *CXCL14* | | Displays chemotactic activity for monocytes but not for lymphocytes, dendritic cells, neutrophils or macrophages. It has been implicated that this cytokine is involved in the homeostasis of monocyte-derived macrophages rather than in inflammation. |
| Chemokine (C-X-C motif) ligand 2 | | *CXCL2* | | Produced by activated monocytes and neutrophils and expressed at sites of inflammation. |
| Chemokine (C-X-C motif) ligand 3 | | *CXCL3* | | Chemotactic activity for neutrophils. May play a role in inflammation and exert its effects on endothelial cells in an autocrine fashion. |
| Chemokine (C-X-C motif) ligand 5 | | *CXCL5* | | Potent chemotaxin involved in neutrophil activation. Produced concomitantly with interleukin-8 (IL8) in response to stimulation with either interleukin-1 (IL1) or tumor necrosis factor-alpha (TNFA). |
| Chemokine (C-X-C motif) ligand 6 (granulocyte chemotactic protein 2) | | *CXCL6* | | Chemotactic for neutrophil granulocytes. |
| Chemokine (C-X-C motif) ligand 9 | | *CXCL9* | | Thought to be involved in T cell trafficking. |
| Chemokine (C motif) receptor 1 | | *XCR1* | | Receptor for chemokines SCYC1 and SCYC2. Subsequently transduces a signal by increasing the intracellular calcium ions level. |
| Chemokine (C-X-C motif) receptor 1 | | *CXCR1* | | Receptor for interleukin 8 (IL8). Causes activation of neutrophils via a G-protein. |
| Chemokine (C-X-C motif) receptor 2 | | *CXCR2/*  *IL8RB* | | Receptor for interleukin 8 (IL8) and transduces the signal through a G-protein activated second messenger system. Mediates neutrophil migration to sites of inflammation. |
| **Cytokine Genes and their Receptors** | | | | |
| Interferon, alpha 2 | *IFNA2* | | Produced by macrophages in response to viral infection. | |
| Interleukin 10 | *IL10* | | Cytokine produced primarily by monocytes and to a lesser extent by lymphocytes. pleiotropic effects in immunoregulation and inflammation. It down-regulates the expression of Th1 cytokines, MHC class II Ags, and costimulatory molecules on macrophages. It also enhances B cell survival, proliferation, and antibody production. This cytokine can block NF-kappa B activity, and is involved in the regulation of the JAK-STAT signaling pathway. | |
| Interleukin 10 receptor, alpha | *IL10RA* | | Mediates the immunosuppressive signal of interleukin 10, and thus inhibits the synthesis of proinflammatory cytokines. Activation of this receptor leads to tyrosine phosphorylation of JAK1 and TYK2 kinases. | |
| Interleukin 10 receptor, beta | *IL10RB* | | Coexpression of this and IL10RA proteins has been shown to be required for IL10-induced signal transduction. | |
| Interleukin 13 | *IL13* | | Immunoregulatory cytokine produced primarily by activated Th2 cells. Down-regulates macrophage activity. Critical in regulating inflammatory and immune responses. | |
| Interleukin 13 receptor, alpha 1 | *IL13RA1* | | Binds with low affinity to interleukin-13 (IL13). Mediate the signaling processes that lead to the activation of JAK1, STAT3 and STAT6 induced by IL13 and IL4. | |
| Interleukin 17C | *IL17C* | | Stimulates the release of tumor necrosis factor alpha and IL-1-beta from the monocytic cell line THP-1. | |
| Interleukin 1, alpha | *IL1A* | | Produced by activated macrophages, IL-1 stimulates thymocyte proliferation by inducing IL-2 release, B-cell maturation and proliferation, and fibroblast growth factor activity. IL-1 proteins are involved in the inflammatory response, being identified as endogenous pyrogens, and are reported to stimulate the release of prostaglandin and collagenase from synovial cells. | |
| Interleukin 1, beta | *IL1B* | | Produced by activated macrophages. Mediator of the inflammatory response, and is involved in a variety of cellular activities, including cell proliferation, differentiation, and apoptosis. | |
| Interleukin 1 family, member 10 (theta) | *IL1F10* | | Participate in a network of interleukin 1 family members to regulate adapted and innate immune responses. Binds soluble IL-1 receptor type 1. | |
| Interleukin 36 receptor antagonist | *IL36RN* | | Inhibit the activation of NF-kappaB induced by interleukin 1 family, member 6 (IL1F6). | |
| Interleukin 36, alpha | *IL36A* | | Immune and inflammatory response | |
| Interleukin 37 | *IL37* | | Suppressor of innate inflammatory and immune responses involved in curbing excessive inflammation. Suppresses, or reduces, proinflammatory cytokine production, including IL1A and IL6, as well  as CCL12, CSF1, CSF2, CXCL13, IL1B, IL23A and IL1RN, but spares anti-inflammatory cytokines. | |
| Interleukin 36, beta | *IL36B* | | Stimulates production of interleukin-6 and interleukin-8 as well as a number of matrix metalloproteases. | |
| Interleukin 36, gamma | *IL36G* | | Function as an agonist of NF-kappa B activation through the orphan IL-1-receptor-related protein 2. | |
| Interleukin 22 | *IL22* | | Contributes to the inflammatory response | |
| Interleukin 5 (colony-stimulating factor, eosinophil) | *IL5* | | Main regulator of eosinopoiesis, eosinophil maturation and activation. | |
| Interleukin 5 receptor, alpha | *IL5RA* | | Receptor for interleukin-5. | |
| Interleukin 1 receptor, type I | *IL1R1* | | Receptor for interleukin-1 alpha (IL-1A), beta (IL-1B), and interleukin-1 receptor antagonist protein (IL-1RA). Binding to the agonist leads to the activation of NF-kappa-B. | |
| Interleukin 1 receptor antagonist | *IL1RN* | | This protein inhibits the activities of interleukin 1, alpha (IL1A) and interleukin 1, beta (IL1B), and modulates a variety of interleukin 1 related immune and inflammatory responses. | |
| Interleukin 8 | *IL8* | | CXC chemokine, major mediators of the inflammatory response. Secreted by several cell types, it functions as a chemoattractant that attracts neutrophils, basophils, and T-cells, but not monocytes. also involved in neutrophil activation. | |
| Interleukin 9 | *IL9* | | Stimulates cell proliferation and prevents apoptosis. Regulator of a variety of hematopoietic cells. | |
| Interleukin 9 receptor | *IL9R* | | Activates different signal transducer and activator (STAT) proteins. | |
| Lymphotoxin alpha (TNF superfamily, member 1) | *LTA* | | Member of the tumor necrosis factor family, produced by lymphocytes. Binds to TNFRSF1A/TNFR1, TNFRSF1B/TNFBR and TNFRSF14/HVEM. In its heterotrimeric form with LTB binds to TNFRSF3/LTBR. Also mediates a large variety of inflammatory, immunostimulatory, and antiviral responses, is involved in the formation of secondary lymphoid organs during development and plays a role in apoptosis. | |
| Lymphotoxin beta (TNF superfamily, member 3) | *LTB* | | Inducer of the inflammatory response system and involved in normal development of lymphoid tissue. | |
| Macrophage migration inhibitory factor (glycosylation-inhibiting factor) | *MIF* | | Regulates the function of macrophages in host defense. Counteracts the anti-inflammatory activity of glucocorticoids. | |
| Aminoacyl tRNA synthetase complex-interacting multifunctional protein 1 | *AIMP1* | | Induced by apoptosis. Involved in the control of angiogenesis, inflammation, and wound healing. Negatively regulates TGF-beta signaling. Involved in glucose homeostasis through induction of glucagon secretion. | |
| Secreted phosphoprotein 1 | *SPP1* | | Enhancing production of interferon-gamma and interleukin-12 and reducing production of interleukin-10. | |
| Tumor necrosis factor | *TNF* | | Mainly secreted by macrophages. Regulation of a wide spectrum of biological processes including cell proliferation, differentiation, apoptosis, lipid metabolism, and coagulation. | |
| CD40 ligand | *CD40LG* | | Mediates B-cell proliferation in the absence of co-stimulus as well as IgE production in the presence of IL-4. | |
| **Other genes involved in inflammatory response** | | | | |
| ATP-binding cassette, sub-family F (GCN20), member 1 | | *ABCF1* | | Protein plays a role in enhancement of protein synthesis and the inflammation process and may be regulated by tumor necrosis factor-alpha. |
| B-cell CLL/lymphoma 6 | | *BCL6* | | Is a zinc finger transcription factor. Acts as a sequence-specific repressor of transcription. Shown to modulate the transcription of START-dependent IL-4 responses of B cells. |
| Complement component 3 | | *C3* | | Plays a central role in the activation of complement system and is required for classical and alternative complement pathways activation. It is a mediator of local inflammatory process. |
| Complement component 4A (Rodgers blood group) | | *C4A* | | Is part of the classical activation pathway and a mediator of local inflammatory process. |
| CCAAT/enhancer binding protein (C/EBP), beta | | *CEBPB* | | Is a bZIP transcription factor and is important in the regulation of genes involved in immune and inflammatory responses. It has been shown to bind to regulatory regions of several acute-phase and cytokine genes. |
| C-reactive protein, pentraxin-related | | *CRP* | | Level of this protein in plasma increases greatly during acute phase response to tissue injury, infection, or other inflammatory stimuli. |
| Caspase recruitment domain family, member 18 | | *CARD18* | | Inhibits generation of IL-1-beta by interacting with caspase-1 and preventing its association with RIP2. Down-regulates the release of IL1B. |
| Leukotriene B4 receptor | | *LTB4R* | | Receptor for extracellular ATP > UTP and ADP. Is a receptor for leukotriene B4, a potent chemoattractant involved in inflammation and immune response. |
| Complement component 5 | | *C5* | | Fifth component of complement, which plays an important role in inflammatory and cell killing processes. It is a mediator of local inflammatory process. C5a also stimulates the locomotion of polymorphonuclear leukocytes (chemokinesis) and direct their migration toward sites of inflammation (chemotaxis). Defects in this gene have also been linked to a susceptibility to liver fibrosis. |
| Toll interacting protein | | *TOLLIP* | | Ubiquitin-binding protein that interacts with several Toll-like receptor (TLR) signaling cascade components. |
| **Housekeeping Genes** | | | | |
| Beta-2-microglobulin | | *B2M* | | Used as normalization control |
| Hypoxanthine phosphoribosyltransferase 1 | | *HPRT1* | | Used as normalization control |
| Ribosomal protein L13a | | *RPL13A* | | Used as normalization control |
| Glyceraldehyde-3-phosphate dehydrogenase | | *GAPDH* | | Used as normalization control |
| Actin, beta | | *actb* | | Used as normalization control |

**Supplementary Table 2:** Primer sequences used in validation study

| **Gene Name** | **Accession Number** | **Sequence** | **Product Sizes (bp)** |
| --- | --- | --- | --- |
| CCL4 | NM_002984.2 | 5'-GCTTCCTCGCAACTTTGTGG  5'-GTTTGGAATACCACAGCTGGC | 75 |
| IL8RB | NM_001168298.1 | 5'-GCCTAGAGCTCTGACTACCAC  5'-CCAGCATCACGAGGGAGTTT | 362 |
| IL1F8 | NM_014438.3 | 5'-GCAGCATTAAGCCTGTCACTC  5'-CCTATGTTGGTCCGCATGGAT | 370 |
| IFNA2 | NM_000605.3 | 5'-GCTCACCCATTTCAACCAGTCT  5'-GAGGGCCACCAGTAAAGCAA | 77 |
| IL19 | NM_013371.3 | 5'-TGCATGTCAAGACCCAGAAGA  5'-TCCTTAGCTTGGATGGCTCTTT | 415 |
| IL9 | NM_000590.1 | 5'-TTGGGCATTCCCTCTGACAAC  5'-TTTGGTTGCATGGCTGTTCAC | 178 |
| CCR5 | NM_000579.3 | 5'-GCATAGTATTCTGTGTAGTGGGA  5'-GGCTGCGATTTGCTTCACAT | 737 |

**Supplementary Figure 1.** A substantial overlap in genes with significant differential expression (p ≤ 0.05) and genes with significant correlation (p ≤ 0.05) to the same histological characteristics of NAFLD


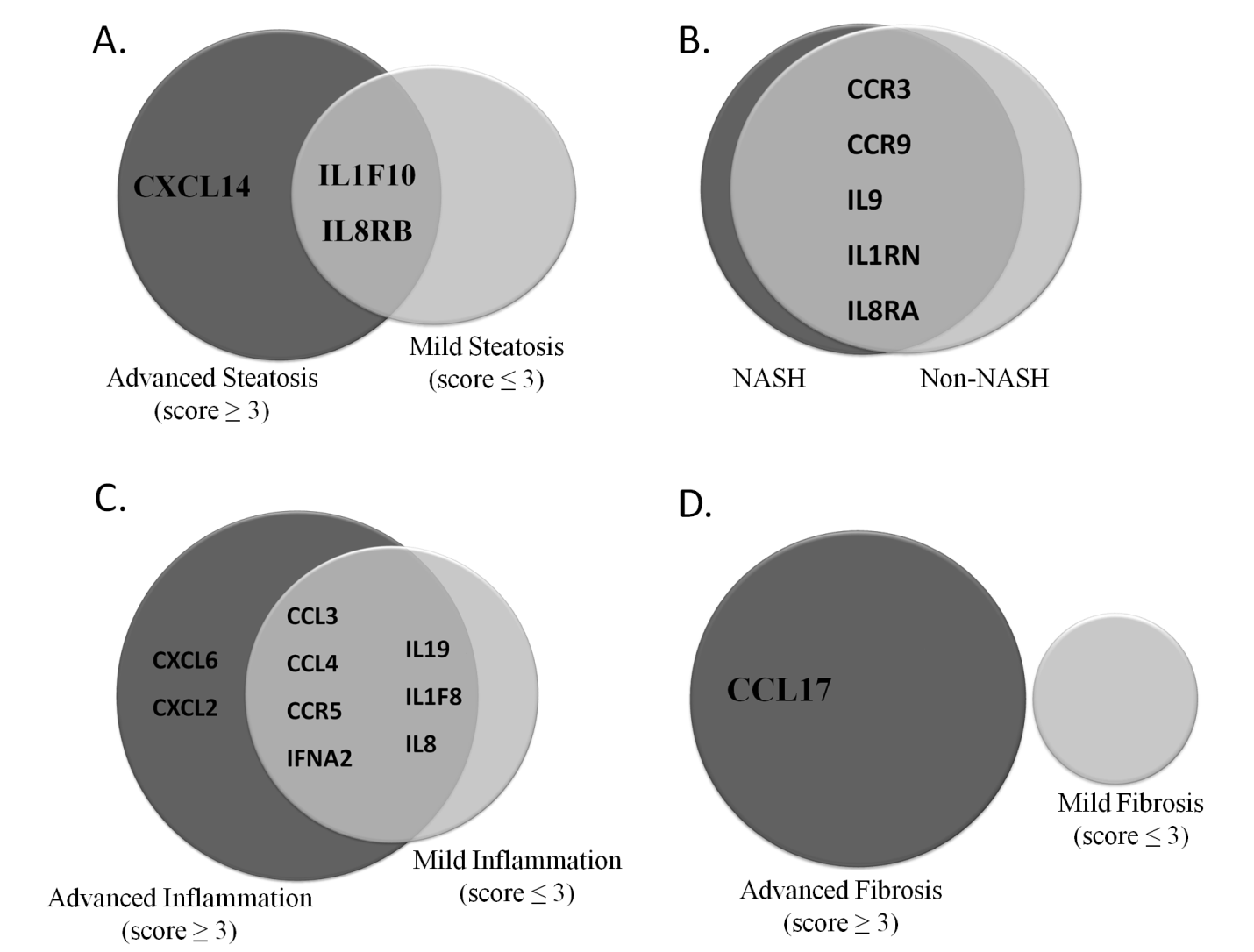

Supplement: Supplementary file 1 — Supplementary Materials contains following Tables and Figure: Supplementary Table 1: Inflammatory cytokines and receptor encoding genes profiled for their expression levels in fundic gastric samples of 20 obese subjects. Supplementary Table 2: Primer sequences used in validation study. Supplementary Figure 1: A substantial overlap in genes with significant differential expression (P ≤ 0.05) and genes with significant correlation (P ≤ 0.05) to the same histological characteristics of NAFLD. [file 684237.f1.docx]
